# Quantitation of TGF-β proteins in mouse tissues shows reciprocal changes in TGF-β1 and TGF-β3 in normal vs neoplastic mammary epithelium

**DOI:** 10.18632/oncotarget.9416

**Published:** 2016-05-17

**Authors:** Kathleen C. Flanders, Yu-an Yang, Michelle Herrmann, JinQiu Chen, Nerissa Mendoza, Amer M. Mirza, Lalage M. Wakefield

**Affiliations:** ^1^ Laboratory of Cancer Biology and Genetics, National Cancer Institute, Bethesda, Maryland, United States of America; ^2^ XOMA Corporation, Berkeley, California, United States of America

**Keywords:** TGF-β isoforms, protein, quantitation, mouse tissues, mammary gland

## Abstract

Transforming growth factor-βs (TGF-βs) regulate tissue homeostasis, and their expression is perturbed in many diseases. The three isoforms (TGF-β1, -β2, and -β3) have similar bioactivities in vitro but show distinct activities in vivo. Little quantitative information exists for expression of TGF-β isoform proteins in physiology or disease. We developed an optimized method to quantitate protein levels of the three isoforms, using a Luminex® xMAP®-based multianalyte assay following acid-ethanol extraction of tissues. Analysis of multiple tissues and plasma from four strains of adult mice showed that TGF-β1 is the predominant isoform with TGF-β2 being ~10-fold lower. There were no sex-specific differences in isoform expression, but some tissues showed inter-strain variation, particularly for TGF-β2. The only adult tissue expressing appreciable TGF-β3 was the mammary gland, where its levels were comparable to TGF-β1. In situ hybridization showed the luminal epithelium as the major source of all TGF-β isoforms in the normal mammary gland. TGF-β1 protein was 3-8-fold higher in three murine mammary tumor models than in normal mammary gland, while TGF-β3 protein was 2-3-fold lower in tumors than normal tissue, suggesting reciprocal regulation of these isoforms in mammary tumorigenesis.

## BACKGROUND

Transforming growth factor-β (TGF-β) proteins are widely expressed and are critical regulators of embryonic development and normal adult tissue maintenance [1]. Dysregulation of the TGF-β pathway is implicated in many diseases including cancer, fibrosis and autoimmunity [2, 3]. Different approaches to therapeutic targeting of TGF-β activity are under development, particularly in fibrotic disorders and cancer [3]. However, little quantitative information is available about the relative levels of TGF-β proteins in normal or diseased tissues due to difficulties in measuring these low abundancy proteins in complex biological samples.

All TGF-βs are synthesized as larger precursor proteins that are cleaved intracellularly, and the upstream pro-regions (known as the “latency-associated peptides” or LAPs) remain non-covalently associated with the mature TGF-βs after secretion. This feature renders them biologically latent until an activation signal is received which releases the mature protein in a form that can bind to the signaling receptors [4]. Mammals express 3 highly conserved TGF-β isoforms, TGF-β1, TGF-β2, and TGF-β3, which have 70-80% identity in the amino acid sequences of their mature active domains [5]. Even though the primary sequences of the mature active isoforms are so similar, there are structural differences between them. NMR studies show that the α3-helical region of TGF-β3 is more disordered than it is in TGF-β1, with TGF-β3 adopting a more “open” flexible conformation in solution, while TGF-β1 predominantly exists in a “closed”, more rigid state [6]. These conformational differences may lead to differential interactions of TGF-β1 and TGF-β3 with unidentified binding proteins, that then affect binding to the signaling receptors [6]. Additionally, TGF-β2 uniquely requires a co-receptor, the type III TGF-β receptor beta-glycan, for presentation to the signaling receptors [7]. Both effects may lead to isoform-specific responses.

While the three TGF-β isoforms generally have similar bioactivities *in vitro* [8], they show distinct activities *in vivo*, as genetic knockout of each isoform generates mice with a unique phenotype (reviewed in [9]). Depending on the background strain, some TGF-β1 null mice die during embryogenesis due to angiogenic defects, while others die shortly after weaning from an autoimmune-like inflammatory disease. TGF-β2 null mice exhibit developmental defects in a number of organs and die just before birth, while TGF-β3 null mice die immediately after birth from an inability to nurse due to cleft palate. These different phenotypes could represent different temporo-spatial patterns of expression of the three isoforms, or they could reflect intrinsically different biological activities. This issue was addressed through generation of mice where the coding sequence of active TGF-β3 was knocked into the TGF-β1 locus with retention of the TGF-β1 LAP, to preserve patterns and levels of expression and activation [10]. Some defects of the TGF-β1 knockout mice were fully rescued by TGF-β3 in the chimeric mice, while others were not, suggesting there may be intrinsic differences in biological activity between the isoforms.

A striking example of TGF-β isoform-specific activities occurs in cutaneous wound healing. In contrast to wounds made in adult tissues, wounds made in mammalian embryos heal without scarring, and this embryonic wound healing is associated with high levels of TGF-β3 and low levels of TGF-β1 and -β2 [11, 12]. When cutaneous wounds made in adult rodents are treated with TGF-β3 they heal with reduced scarring and inflammation [13], suggesting that TGF-β3 can oppose the well-documented pro-desmoplastic effects of TGF-β1 *in vivo*. This raises the issue of whether isoform-selective agents might be more efficacious than pan-TGF-β inhibitors in treating certain diseases, such as those with a prominent fibrotic component.

Given these differences in isoform activity, we felt it was important to accurately determine the quantity of TGF-β isoform proteins present in tissues under both physiologic and pathological conditions. Since both TGF-β1 and -β3 are subject to extensive translational regulation [14, 15], mRNA levels of these isoforms may not correspond directly to protein levels. While the quantitation of TGF-βs in simple biological samples such as serum-free cell-conditioned media is straightforward, the generally low levels of TGF-β in tissues pose problems for accurate quantitation due to the high background of more abundant cellular proteins, and the propensity of this highly hydrophobic protein to bind non-specifically to other macromolecules and inorganic surfaces. Furthermore, TGF-β is synthesized and secreted as a biologically latent form [4] which must be activated to be detectable in existing bioassays and immunoassays. Many detergent-based tissue extraction methods do not activate TGF-β adequately, and when tissue extracts are transiently treated with acid to make the TGF-β detectable there is often loss of protein through precipitation.

The first step in the original isolation and purification of TGF-β protein from tissues was acid-ethanol extraction [16]. This method allows extraction of acid-stable proteins in high yield and free of the bulk of the other tissue constituents. TGF-β isoform proteins have been quantitated in such tissue extracts using custom sandwich ELISA assays [17, 18]. The development of Luminex® xMAP®-based multianalyte profiling technology allows simultaneous quantitation of the three TGF-β isoforms in a sample. Here we show that the acid-ethanol extraction step, while labor-intensive, is necessary for accurate quantitation of total TGF-β protein in tissue extracts and we establish an optimized protocol. We quantitated the TGF-β isoforms in normal mouse tissues and plasma, assessing strain- and sex-specific differences, to establish baseline expression in adult tissues. We also identified novel isoform-specific changes in TGF-β expression in three mouse mammary tumor models.

## RESULTS

### Optimization of extraction method

**Figure 1:**
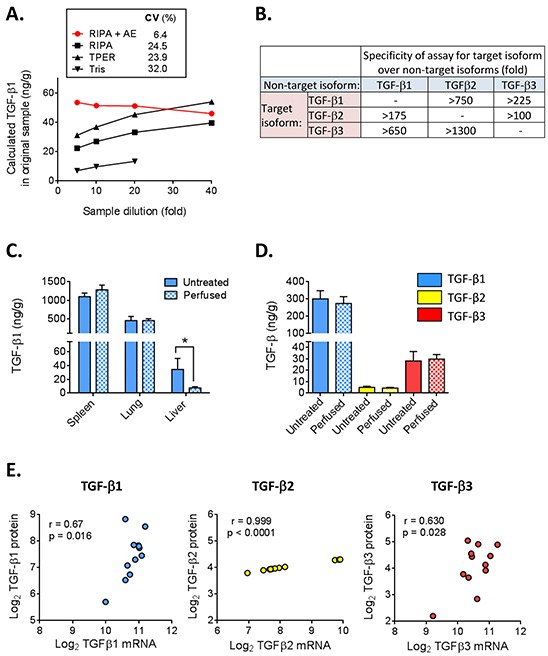
Optimization and characterization of TGF-β 1, 2, and 3 multiplex assay for tissue extracts **A.** Frozen mouse kidney was lysed in RIPA, T-PER or Tris buffer and a portion of the RIPA extract was further treated with acid-ethanol (AE), dialyzed and lyophilized as described in Methods. TGF-β1 levels were measured in extracts prepared by the different methods at 4 different sample dilutions. The calculated TGF-β1 concentration in the original tissue sample is plotted for each dilution. Ideally, results should be independent of sample dilution. CV (%) represents the coefficient of variation across the dilutions for each extraction method. **B.** Estimation of assay specificity using a high concentration (50 ng/ml TGF-β1, 25 ng/ml TGF-β2 and 92 ng/ml TGF-β3) of the purified non-target TGF-β isoforms to assess cross-reactivity. **C.** TGF-β1 levels in tissues isolated from untreated or transcardially-perfused BALB/c mice. Values are mean ± standard deviation (n=4/tissue) *p<0.05. **D.** TGF-β isoform levels in 4T1 mammary tumors harvested from mice that underwent cardiac perfusion or mice that were not perfused. Values are mean ± standard deviation (n=5). **E.** Correlation of TGF-β isoform protein levels with TGF-β mRNA levels for 12 different murine tumor models. Results are plotted as the median value of each parameter for each model. The Pearson correlation coefficient (r) and p-value for the correlation are given.

Acid-ethanol extraction was the first step used in the original isolation and purification of TGF-β from tissues [16, 19]. This method minimizes proteolytic activity and extracts acid-stable proteins in high yield [16], but it is a labor-intensive, multi-step protocol. Furthermore, homogenization of frozen tissue directly into acid-ethanol caused protein to precipitate on the outside of the tissue fragments. To determine whether there might be a better extraction method for archived frozen tissue samples, we homogenized frozen kidneys directly in RIPA buffer, T-PER buffer or Tris-HCl (pH 7.5). Similar methods have previously been used to prepare tissues for TGF-β measurements [20–22]. A portion of the RIPA homogenate was then further extracted with acid-ethanol, dialyzed, lyophilized and resuspended as described in Materials and Methods. The amount of TGF-β1 present in the original sample was determined using the Luminex-based multiplex TGF-β assay on serial dilutions of the various extracts (Figure 1A). Extraction with Tris-HCl showed significantly lower amounts of TGF-β than extraction with either RIPA or T-PER. Furthermore, dilution of samples extracted with RIPA or T-PER alone resulted in an increase in the calculated ng TGF-β1/g tissue (coefficient of variation from 24-32% over an 8-fold dilution range), suggesting there are sample matrix effects that generate systematic errors in quantitation with these methods. Acid-ethanol extraction of the RIPA homogenate, followed by dialysis for solvent exchange, stabilized the TGF-β1 value (coefficient of variation of 6.4%), making results independent of sample dilution. Since TGF-β levels differ widely between tissues (see later), we believe the acid-ethanol step is necessary for accurate TGF-β quantitation, as different tissues must be assayed at very different dilutions to fall within range of the standard curve. Without the acid-ethanol step, the matrix effects in the sample extracts are most pronounced at lowest sample dilutions and will have a disproportional impact on samples with low TGF-β expression. The amount of TGF-β1 in frozen kidney homogenized in RIPA and extracted in acid-ethanol (38.2 ng/g tissue) was similar to that found in fresh kidney extracted directly into acid-ethanol (42.4 ng/g tissue) suggesting that prior homogenization in RIPA buffer overcomes the sample losses previously associated with direct homogenization of frozen tissues in acid-ethanol.

Platelets are the most concentrated source of TGF-β1 *in vivo* and contain approximately 40-100x more TGF-β1 than most cells [23]. To determine if residual blood present in tissue samples would contribute significantly to TGF-β levels, normal spleen, lung and liver, as well as tumors from orthotopically-implanted 4T1 mammary carcinoma cells were harvested either with or without cardiac perfusion of the mice with PBS to clear residual blood from all tissues. Figure 1C shows that TGF-β1 levels were similar in perfused and non-perfused spleen and lung, but were significantly decreased in perfused liver. The liver contains 10-15% of the body's blood volume and removal of this blood with its high concentration of TGF-β1 largely accounts for the reduced amount of TGF-β1 in the perfused liver. Mammary tumors contain relatively high levels of all 3 TGF-β isoforms which were not significantly altered by perfusion (Figure 1D). Since perfusion will be impractical in many experimental settings, mice were not perfused before tissue isolation in our subsequent studies. However, if organs with a particularly high blood volume and a low intrinsic TGF-β1 content are the focus of a study, perfusion should probably be performed.

### Detection limits, specificity and correlation of protein with mRNA levels

The detection limits of the assays were 0.6, 0.3 and 1.0 ng/g tissue for TGF-β1, TGF-β2 and TGF-β3 respectively, while the detection limits for plasma were 72, 35 and 120 pg/ml. Thus, the TGF-β3 assay is approximately two-fold less sensitive than the TGF-β1 and TGF-β2 assays. Using purified TGF-β isoforms, we tested the specificity of the assays for each target isoform (Figure 1B). The cross-reactivity of TGF-β1 with assays for TGF-β2 and TGF-β3 is particularly important to assess because of the much higher abundance of the TGF-β1 isoform. A high level of specificity was observed, with cross-reactivity with the TGF-β1 isoform being <0.6% in the TGF-β2 assay and <0.15% in the TGF-β3 assay.

To determine to what extent TGF-β protein levels correlate with mRNA levels, we compared TGF-β isoform mRNA levels determined using Affymetrix microarrays (4 tumors/model) with protein levels assessed by acid-ethanol extraction (5 independent tumors/model) for a total of 12 different mouse tumor models representing a range of TGF-β levels, and plotted the median values for each model and approach (Figure 1E). The correlation between mRNA and protein levels for TGF-β2 was high, but large increases in TGF-β2 mRNA were associated with very small increases in protein levels. In contrast, mRNA and protein were more weakly correlated for TGF-β1 and TGF-β3, but the relationship between increases in protein and increases in mRNA gave slopes closer to 1. Overall, the data suggest that transcript levels are not a very reliable surrogate for protein levels.

### Expression of TGF-β 1, 2, and 3 in adult mouse tissues

**Figure 2:**
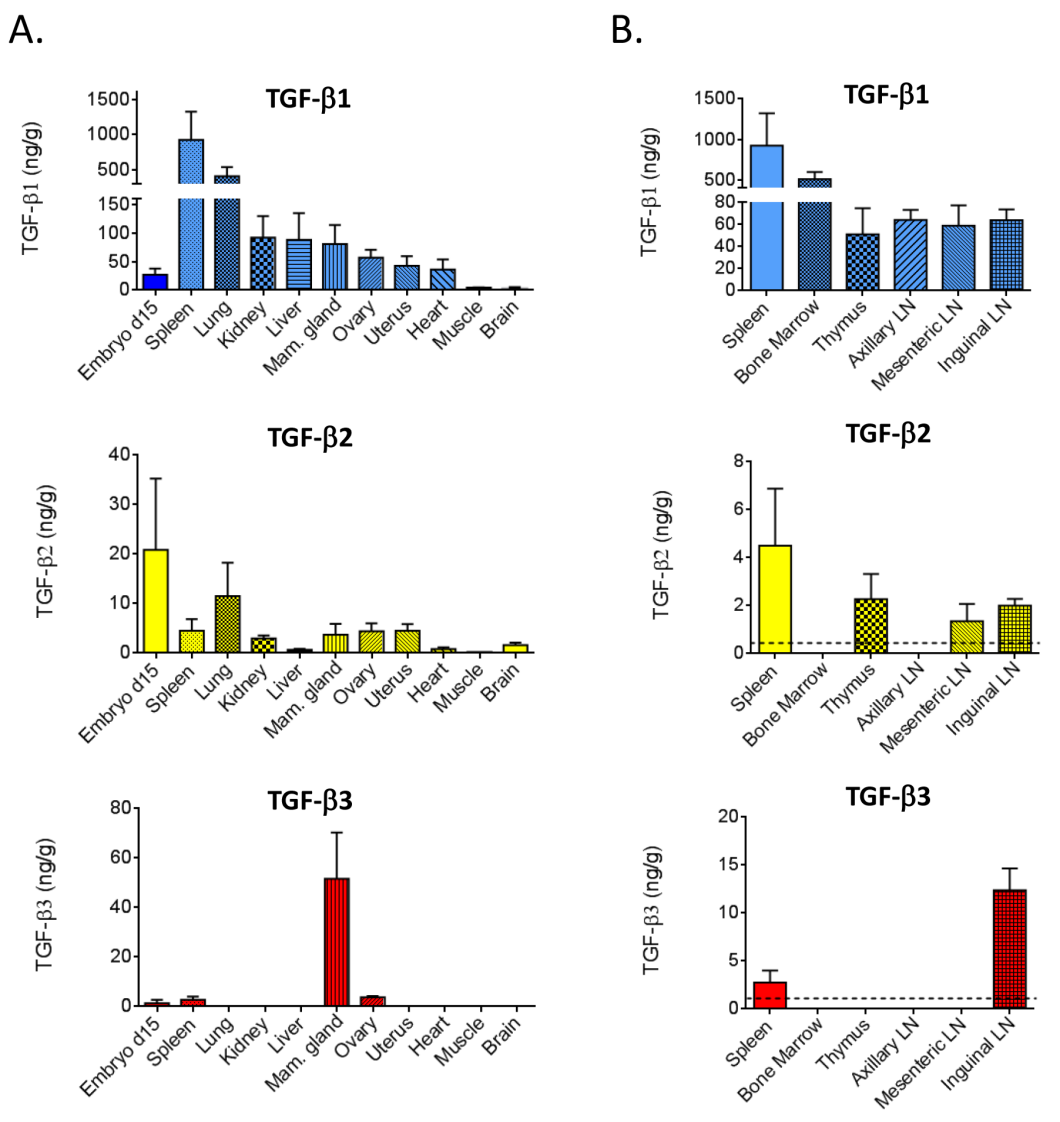
TGF-β isoforms in tissues from BALB/c mice **A.** TGF-β1, 2, and 3 were quantitated by multiplex assay from acid-ethanol extracts of tissues from 9 wk old female BALB/c mice, or from 15 d gestation BALB/c embryos. Values are mean ± standard deviation (n=4/tissue). **B.** In an independent experiment, select immune/hematopoietic tissues were assessed as in (A). The dotted lines indicate the assay detection limit.

To establish baseline TGF-β levels, we used 9 week old adult female BALB/c mice, with day 15 gestation embryos included for comparison (Figure 2A). Spleen contained the highest levels of TGF-β1 (~900 ng/g tissue). Next came lung (~400 ng/g), followed by kidney, liver, mammary gland, ovary, uterus and heart (40-100 ng/g), with the lowest levels seen in muscle and brain (2-4 ng/g). Detectable amounts of TGF-β2 were found in all of these tissues, but the levels were approximately 10-100-fold lower than those of TGF-β1. In contrast, TGF-β3 was only detectable in the spleen, ovary and mammary gland. The expression of TGF-β3 in the mammary gland was particularly striking with TGF-β3 levels (52 ng/g) being comparable to TGF-β1 (80 ng/g). While embryonic levels of TGF-β1 were lower than seen in most adult tissues, embryonic TGF-β2 levels were higher than those in any adult tissues, and TGF-β3 was detectable at a low level in the embryo, whereas it was not detected in most adult tissues.

Given the importance of TGF-βs in immune regulation [24], we assessed TGF-β isoforms in additional hematopoietic and lymphoid tissues of young adult female BALB/c mice (Figure 2B). Like the spleen, the bone marrow had high levels of TGF-β1 (~400 ng/g), but levels of TGF-β2 or TGF-β3 were undetectable. Thymus and lymph nodes at three different locations expressed 40-60 ng/g of TGF-β1 and ~2 ng/g of TGF-β2, although the axillary lymph nodes were outliers in having no detectable TGF-β2. TGF-β3 was only detectable in the inguinal lymph node. Since this lymph node is located in the mammary gland which is the only adult organ expressing high levels of TGF-β3 protein, the TGF-β3 in the inguinal lymph node may reflect contamination with mammary epithelium, or accumulation of TGF-β3 that drains from the mammary gland.

**Figure 3:**
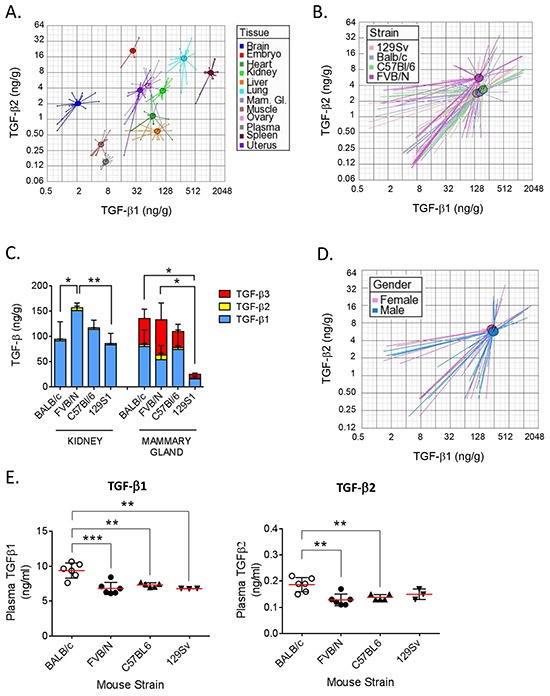
Comparison of TGF-β isoform levels in 4 mouse strains **A.** Visualization of differences in TGF-β1 and TGF-β2 expression in nine individual tissues and plasma. Values for the day 15 gestation BALB/c embryo and adult BALB/c ovary are shown for comparison. Circles represent the centroids for each tissue, and rays connect to individual data points for replicate tissue samples (n=4) from all four strains. Centroids for ovary and mammary gland are overlapping. Please note the differences in the scale of the x and y axes. **B.** Visualization of overall differences in TGF-β1 and -β2 between 4 strains of mice. Circles represent the centroids for each strain and rays connect to data points for individual tissue samples for each strain. Data from all nine tissues and plasma (n=4 replicates/tissue/strain) were used to calculate the strain centroids. Please note the differences in the scale of the x and y axes. **C.** Stackplots showing levels of TGF-β1 (blue), TGF-β2 (yellow), and TGF-β3 (red) in kidney and mammary gland (n=4 per tissue) in 9 wk old female mice from 4 different strains. Values are mean ± standard deviation. p-values are for differences in total TGF-β levels between strains. *p<0.05,** p<0.01; one-way ANOVA with Tukey's multiple comparison test. **D.** Visualization of overall differences in TGF-β1 and -β2 levels between male and female FVB/N mice. Circles represent the centroids for each sex, and rays connect to data points for individual tissue samples for each sex. Data from seven tissues and plasma (n=4 replicates/tissue/sex) were used to calculate the centroids. Please note the differences in the scale of the x and y axes. **E.** Levels of TGF-β1 and -β2 in plasma from 4 mouse strains (n=6). Values are mean ± standard deviation. ** p<0.01, ***p<0.001; one-way ANOVA with Tukey's multiple comparison test.

It has been suggested that the different propensity of different mouse strains to develop certain pathologies such as fibrosis may reflect interstrain differences in endogenous levels of TGF-β isoforms [25]. TGF-β isoforms in BALB/c, FVB/N, C57BL/6 and 129S1 strains were compared. A composite view of the TGF-β1 and TGF-β2 levels across 10 tissues and plasma is given graphically in Figure 3A. The circles represent the centroid (geometric mean) for each tissue and the rays connect to individual data points for replicate samples from the four mouse strains. This visualization shows that, in general, tissue-specific differences are bigger than strain-specific differences, though the sex-hormone responsive tissues, mammary gland, uterus and ovary all showed similar TGF-β1 and TGF-β2 levels. Plotting the all-tissue centroids for each strain, showed relatively little difference in overall TGF-β1 levels between mouse strains, though FVB/N mice had ~2x higher overall TGF-β2 levels than the other strains (Figure 3B). However significant inter-strain differences are seen when individual tissues are considered (Supplementary Tables S1–S3). For example, FVB/N mice have significantly more total TGF-β (all three isoforms combined) in the kidney than the other strains, while 129S1 mice have significantly less total TGF-β in the mammary gland compared with the other strains (Figure 3C). We found no significant gender-related differences in tissue TGF-β levels in age-matched male FVB/N mice (sex-specific centroids for all tissues in Figure 3D and individual data in Supplementary Figure S2 and Supplementary Table S4).

TGF-β can be measured in plasma without acid-ethanol extraction because of the lower total protein concentration, although plasma samples must still be transiently acid-activated prior to assay. Normal plasma TGF-β1 levels in 9 wk old female mice were in the range of 7-10 ng/ml when care was taken to minimize platelet degranulation (Figure 3E). TGF-β1 levels were ~40% higher in BALB/c mice compared to the other strains (p<0.001). Plasma levels of TGF-β2 were ~50-fold below those of TGF-β1 and followed the same pattern, with BALB/c mice showing the highest levels. No TGF-β3 protein was detected in any plasma samples (detection limit = 0.12 ng/ml).

### The normal mammary gland contains high levels of TGF-β3 protein

**Figure 4:**
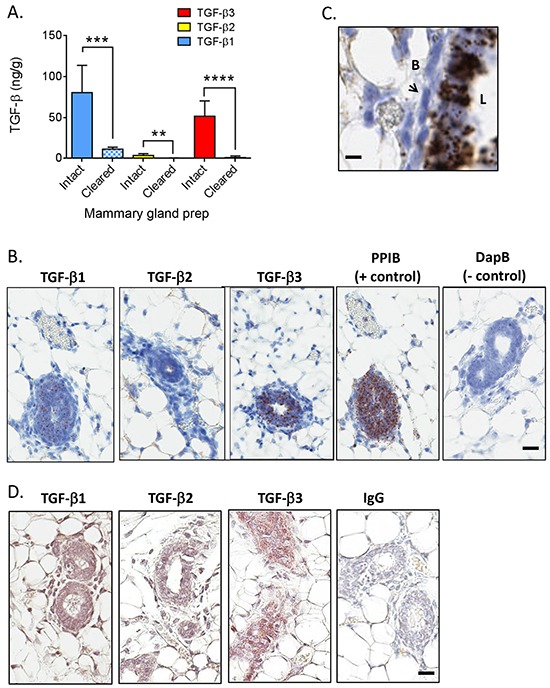
TGF-β isoform expression in the normal mammary gland of BALB/c mice **A.** Levels of TGF-β1 (blue), TGF-β2 (yellow), and TGF-β3 (red) in intact (solid bars) or cleared (cross-hatched bars) mammary glands from BALB/c mice (n=4 for intact and n=7 for cleared). Values are mean ± standard deviation. **p<0.01, ***p<0.001, ****p<0.0001; unpaired t-test. **B.** *In situ* hybridization using probes for the indicated molecules in a virgin mammary gland from an adult BALB/c mouse. Brown dots indicate positive signal. Cyclophilin B/PPIB and DapB (bacterial dihydropicolinate reductase) were used as positive and negative controls. Bar = 25 μm. **C.** Higher magnification view of *in situ* hybridization for TGF-β3 in a virgin mammary gland from an adult BALB/c mouse. “L” is the luminal side of the gland and “B” is the basal side. Arrow points to a myoepithelial cell. Bar = 10μm. **D.** Immunohistochemistry using antibodies for the indicated molecules in a virgin mammary gland from an adult BALB/c mouse. Brown staining indicates positive signal. Normal rabbit IgG (IgG) was used as a negative control. Bar = 25 μm.

The mammary gland was the only normal adult tissue examined with substantial TGF-β3 expression, and it was the only tissue where the quantity of any other TGF-β isoform was comparable to that of TGF-β1. Mammary fatpads pre-cleared of epithelium contained almost no TGF-β3 (Figure 4A), suggesting that the epithelium is the major location of this isoform. There were also significant reductions in TGF-β1 and TGF-β2 protein in the cleared mammary fatpad compared with the intact gland. TGF-βs are secreted proteins that bind to many extracellular matrix components and can be sequestered from a variety of cellular sources [4]. To determine which cell types synthesize the TGF-β isoforms, we performed *in situ* hybridization. All three TGF-β isoform mRNAs were found predominantly in the luminal epithelium of the mammary ducts of 9 wk old virgin BALB/c mice (Figure 4B) with higher magnification for TGF-β3 shown in Figure 4C. Direct quantitative comparison between the different isoforms by this technique cannot be made because of the differing efficiencies of the *in situ* hybridization reactions. Mammary glands from FVB/N mice showed similar patterns of TGF-β isoform mRNA expression (Supplementary Figure S3). Immunohistochemistry confirmed that protein for each isoform was present in the mammary epithelium of BALB/c mice (Figure 4C), but TGF-β1 protein was also readily detectable in mammary stromal cells, consistent with the presence of extractable TGF-β1 protein in the cleared mammary fat pad (Figure 4A).

### TGF-β1 protein is upregulated and TGF-β3 protein is downregulated in mammary gland tumors

**Figure 5:**
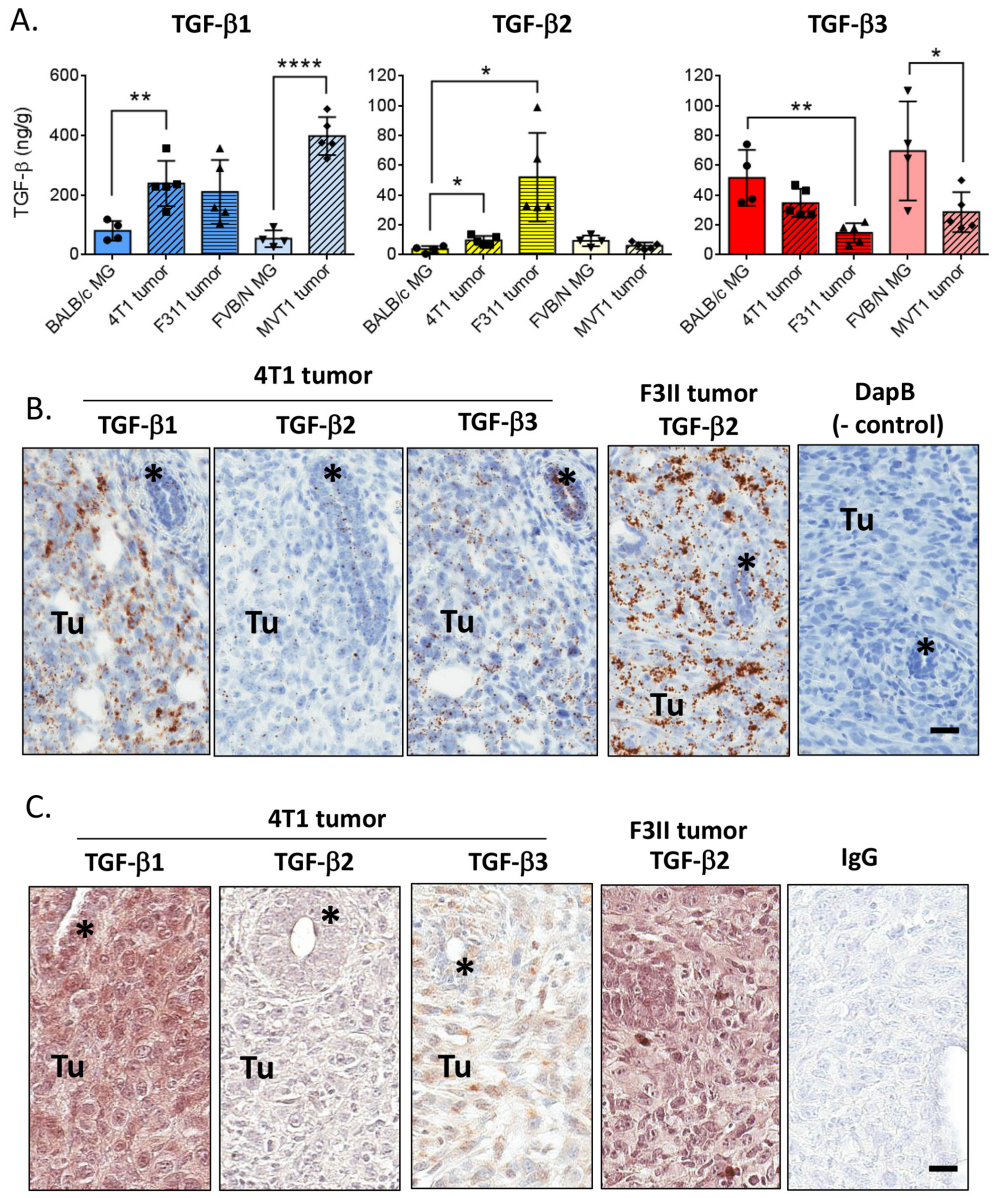
TGF-β isoform expression in mouse mammary tumors **A.** Quantitation of TGF-β1, 2, and 3 protein in mouse mammary tumors compared with the normal mammary gland of the appropriate strain. Values are mean ± standard deviation. *p<0.05, **p<0.01, ****p<0.0001; unpaired t-test vs normal mammary gland. **B.** *In situ* hybridization using probes for the indicated molecules in 4T1 and F311 mammary tumors. “Tu” marks area of tumor while * marks an engulfed mammary duct. Cyclophilin B/PPIB and DapB (bacterial dihydropicolinate reductase) were used as positive and negative controls. Bar = 25 μm **C.** Immunohistochemistry using antibodies for the indicated molecules in 4T1 and F311 mammary tumors. “Tu” marks area of tumor while * marks an engulfed mammary duct. IgG indicates the negative control. Bar = 25 μm.

Given the high levels of TGF-β3 in the mammary gland, we assessed whether this unique expression pattern was maintained in mouse mammary tumors (Figure 5A). TGF-β1 protein was ~3x higher in orthotopically-implanted tumors from 4T1 and F311 breast cancer models (BALB/c strain), and ~8x higher in MVT1 tumors (FVB/N strain) when compared to the normal mammary gland of the matched strain. TGF-β2 levels were also increased in the 4T1 and F3II tumors, but were unchanged in the MVT1 tumors. In striking contrast, TGF-β3 protein levels were 2-3-fold lower in tumors than in the normal mammary gland.

These results were confirmed by *in situ* hybridization (Figure 5B). As was seen at the protein level, in the 4T1 tumors TGF-β1 mRNA is more highly expressed in the tumor cells than in the normal ductal epithelium of an engulfed duct, while TGF-β3 mRNA expression remains high in the normal mammary duct and is reduced in the tumor cells. TGF-β1 mRNA is also expressed in endothelial and immune cells in the tumor. TGF-β2 mRNA is low but evident in both the tumor and normal mammary duct. The F311 tumor model showed similar mRNA expression for TGF-βs 1 and 3, but had much higher expression of TGF-β2 mRNA in the tumor (Figure 5B). These isoform expression patterns were less apparent when assessed at the protein level by immunohistochemistry, possibly due to diffusional spreading of the secreted protein (Figure 5C).

## DISCUSSION

Here we present the first study to systematically quantitate TGF-β1, TGF-β2, and TGF-β3 protein levels in tissues in the normal adult mouse. As TGF-β pathway antagonists are moving into clinical trials, this study provides baseline information to help in addressing the potential desirability and consequences of isoform-specific therapeutic neutralization of TGF-β. The need for improved methods to quantitate TGF-β proteins was highlighted recently by a large cancer tissue microarray study showing that TGF-β immunohistochemistry results did not correlate well with TGF-β levels assessed by mRNA quantitation or Western blotting [26], thereby posing challenges for how best to stratify patients for clinical trials with anti-TGF-β therapies. We have refined the existing methodology for TGF-β protein quantitation and made a number of important findings as discussed below.

We found that an acid-ethanol extraction step was critical to recover the maximal amount of TGF-β protein from tissues and to accurately quantitate it across a range of sample dilutions. Although this step is labor intensive, we have not found a way of eliminating this requirement while still generating robustly reproducible data. Since acid-ethanol extraction activates latent TGF-β, it is levels of total (latent plus bio-active) TGF-β that are quantitated by this approach. Bead-based multiplexed protein detection technology allows all three TGF-β isoforms to be assayed simultaneously which increases the accuracy of inter-isoform comparison. In a limited comparison of TGF-β protein levels with mRNA levels as determined by Affymetrix microarray in mammary tumors, we found discrepancies between levels of protein and mRNA levels for all TGF-β isoforms confirming the importance of determining protein levels.

In adult tissues (with the exception of mammary gland) TGF-β1 was the predominant isoform, with TGF-β2 and TGF-β3 levels being approximately 10-100-fold lower. TGF-β1 was more abundant in adult tissues than in the 15 day embryo, while the reverse was true for TGF-β2 and TGF-β3. TGF-β1, 2 and 3 mRNA and protein are expressed throughout embryonic development [27, 28]. Given the embryonic or peri-natal lethality of the TGF-β2 and TGF-β3 knockout mice [29, 30], together these findings are consistent with the concept that TGF-β1 plays important roles in adult tissue homeostasis, while TGF-β2 and TGF-β3 function as major players in development. The overall lower levels of TGF-β3 than TGF-β2 protein observed in extracts from whole 15d embryos may reflect findings from immunohistochemical and functional studies suggesting that TGF-β3 is highly localized within specialized regions of developing tissues [31, 32].

In the adult animals, we saw major differences in TGF-β1 and TGF-β2 expression between tissues. The high levels of TGF-β1 in spleen and bone marrow probably derive primarily from megakaryocytes, the precursors of platelets which are the most concentrated source of TGF-β1 *in vivo* [23]. In contrast, TGF-β2 was highest in the lung, but still much lower than TGF-β1, a pattern that was consistent across all tissues. There were no significant sex-specific differences in TGF-β expression.

Differences in TGF-β levels have been proposed to underlie differences across mouse strains in susceptibility to some diseases [33]. For example, differences in TGF-β1 gene expression in skin in two mouse strains may alter skin tumor susceptibility [34]. Aggregating results from all tissues, showed that the FVB/N strain had ~2x higher TGF-β2 overall than the other strains, while overall TGF-β1 levels were similar among strains. This means that no one mouse strain can be considered to be a “high TGF-β strain” as has been previously suggested. However, there were significant strain-specific differences in some individual tissues. TGF-β1 has been implicated in the pathogenesis of fibrotic diseases [35], and mouse strains differ in their fibrotic responses in an organ-specific manner [36] so we were interested to see if there was correlation between TGF-β1 levels and fibrosis susceptibility in target organs. For example, BALB/c mice are resistant to pulmonary fibrosis but sensitive to hepatic fibrosis, while C57BL/6 mice show the reverse sensitivity [36]. Our data show that baseline TGF-β1 levels are not significantly different between strains in either organ, suggesting either that differences in TGF-β protein expression occur after the fibrogenic insult [25], or that down-stream factors at the level of latent TGF-β activation, signaling or signal interpretation contribute to the difference in fibrosis between these strains [37].

Circulating TGF-β levels are difficult to measure because of potential contamination by platelet-derived TGF-β1, but unlike tissues, plasma samples do not require acid-ethanol extraction for accurate quantitation. Using retro-orbital bleeding and taking care to minimize platelet degranulation we found plasma levels of TGF-β1 to be ~7 ng/ml in FVB/N, C57BL/6 and 129S1 mice, and ~40 % higher in BALB/c mice (~10ng/ml). These values are in good agreement with others in the literature [38, 39] and well below that of TGF-β1 in mouse serum (136 ng/ml in C57BL/6 mice) [22]. Others have also reported no significant differences in plasma TGF-β1 levels between C57/BL6 and 129S1 strains [40]. A novel method that obtains free-flowing blood by percutaneous puncture of the left cardiac ventricle under ultrasound guidance further reduces platelet degranulation and results in plasma levels of ~2 ng/ml TGF-β1 in mice [39]. Thus our values may still overestimate true systemic circulating TGF-β1 levels. For the other two isoforms, we measured 0.1-0.2 ng/mL of TGF-β2 in plasma and no detectable TGF-β3 (< 0.1 ng/mL). While mouse plasma levels of TGF-β2 and 3 have not been previously reported, our results are consistent with those in human where the average TGF-β1 level is 4 ng/mL, but TGF-β2 and 3 levels are extremely low (<0.3 and 0.1 ng/ml, respectively) [41].

Our study is the first to quantitate TGF-β3 protein in adult mouse tissues. Aside from the spleen and the ovary which both had a low level of TGF-β3, the virgin mammary gland was the only adult organ examined to show substantial TGF-β3 expression (up to 80 ng/g). Across mouse strains, TGF-β3 comprised ~30-50% of the total TGF-β protein in the mammary gland, and *in situ* hybridization revealed that the mammary luminal epithelium is the major source of all 3 isoforms. This finding is intriguing as the mammary gland is an organ that uniquely undergoes most of its development post-natally, including ongoing cycles of remodeling with pregnancy, and to a lesser extent during the estrous cycle [42]. Dramatic differences in TGF-β3 mRNA and protein expression are seen depending on the functional state of the gland (reviewed in [43]). TGF-β3 levels increase in the ducts and alveoli during pregnancy, decrease during lactation and then transiently increase during involution before decreasing again as the mammary gland returns to its pre-partum state [44]. Given that TGF-β3 prevents pathological scarring in cutaneous wound healing while TGF-β1 tends to induce scar formation [13], we have speculated that one role for TGF-β3 in the mammary gland may be to limit stromal activation and prevent scarring [43]. This feature would allow repeated cycles of lactation and involution to occur without compromising mammary function or inducing a sustained pro-inflammatory reaction that could promote tumorigenesis. Indeed, elevated TGF-β3 levels in the post-partum rat and mouse mammary glands have been associated with pregnancy-induced protection against mammary cancer [45].

Elevated TGF-β1 levels have been associated with poor outcome in human breast cancer [46–48] and TGF-β1 is thought to function primarily as a pro-progression factor in late-stage disease [49, 50]. However, the situation for TGF-β3 is less clear and correlative data suggest that TGF-β3 may be protective. TGF-β3 contributes to a 70-gene signature which predicts clinical outcome in breast cancer, with high expression of TGF-β3 mRNA being associated with a reduced risk of metastasis [51]. Analysis of publically available clinical breast cancer microarray datasets shows a negative correlation between TGF-β3 mRNA levels and increasing tumor grade [43], and in a large population-based breast cancer cohort, TGF-β3 protein as assessed immunohistochemically, was lower in poorly differentiated tumors than in well- or moderately-differentiated tumors [52].

We demonstrated for 3 murine mammary tumor models that TGF-β1 protein expression increases in the tumor when compared to the normal mammary gland, while TGF-β3 expression decreases. These changes are consistent with the possibility that these two TGF-β isoforms may play opposing roles during tumor progression. A gene expression signature enriched for genes involved in the wound-healing response predicts increased risk of metastasis and death in breast, lung and gastric cancers [53, 54]. Since tumors have been described as “wounds that do not heal” [55], and TGF-β3 opposes the actions of TGF-β1 in normal wound healing [13], the high expression of TGF-β3 in breast cancer progression may be protective through suppression of aberrantly engaged or persistent wound healing responses. If similar results are seen in human clinical samples, our demonstration of increased TGF-β1 protein and decreased TGF-β3 protein in the mammary tumors raise the possibility that TGF-β pathway antagonists that spare TGF-β3 may have advantages over pan-isoform-specific inhibitors in breast cancer therapy. Our observations also raise interesting questions regarding the role of these two TGF-βs in early vs late disease, the importance of absolute amounts of the isoforms vs ratios of the two, and issues of isoform cross-regulation. Furthermore, since these isoforms can be activated by different mechanisms [56], the change in balance between TGF-β1 and TGF-β3 may sensitize or desensitize the system to certain activation signals.

While we have found acid-ethanol extraction to be the best sample preparation method for robust, reproducible measurement of TGF-β protein in tissues, this approach suffers from a number of drawbacks that need to be addressed by future technology developments. Firstly, it is time-consuming and labor-intensive, and would not adapt readily to routine clinical use. Secondly, it measures total (latent plus biologically active) TGF-β, whereas the most biologically relevant pool of TGF-β is the active fraction. *In vivo* activation of latent TGF-β is a tightly regulated process that is believed to be one of the major mechanisms for controlling the diverse bioactivities of TGF-β [4]. Activation is mediated by a variety of mechanisms that include interactions with thrombospondin, certain proteases and integrins, or physicochemical activation by low pH or reactive oxygen species [4]. Detection of active TGF-β is challenging due to the very low levels present under most conditions, and the ready loss of the active fraction on handling. Sensitive bioassays have been used for this purpose, in conjunction with TGF-β isoform-specific neutralizing antibodies [57, 58], but such assays are prone to interference by other sample components. An elegant immunofluorescent method for detection of active TGF-β *in situ* has been described [59], but it is not quantitative. Finally, novel sources of active TGF-β are being identified, such as on the surface of exosomes [60], and these need to be incorporated into our thinking about how samples should be best prepared for TGF-β measurement. The ability to reliably quantitate active TGF-β sensitively and specifically in its various forms would represent a major next step forward in the field.

In summary, we have described an optimized method for accurate assessment of total levels of the three TGF-β protein isoforms in tissues. We established baseline levels in normal mouse tissues and performed a preliminary exploration of changes in isoform expression in mammary tumorigenesis. Since the TGF-βs are so highly conserved across species, the assay should be applicable to human samples. It will be interesting to determine whether similar inter-tissue and inter-individual differences in isoform expression are also seen in humans.

## MATERIALS AND METHODS

### Ethics statement

Animal experiments were approved by the National Cancer Institute Animal Care and Use Committee and were in conformity with national guidelines for the care and use of laboratory animals.

### Collection of mouse tissues and plasma

Tissues were harvested from 9 wk old male and female FVB/N mice, as well as, female C57BL/6, 129S1, and BALB/c mice. Mice were obtained from National Cancer Institute-Frederick Animal Production Area (FVB/N, C57Bl/6 and BALB/c) or Jackson Labs (129S1). A minimum of four mice/group were used. Mice were euthanized by CO_2_ inhalation and lung, heart, kidney, spleen, liver, femoral muscle, brain, uterus and mammary gland (#4) were harvested and snap-frozen in liquid N_2_. For BALB/c mice, ovary, bone marrow, thymus and axillary, mesenteric and inguinal lymph nodes were also harvested. Two to four lymph nodes were pooled for analysis. Embryos (~15 d gestation) were isolated from a pregnant BALB/c mouse and extracted without further dissection. To address the distribution of TGF-β isoforms in different cellular compartments of the mammary gland, the mammary fat-pad was cleared of mammary epithelium by surgically removing a portion of the #4 mammary gland, proximal to and including the lymph node, in 3 wk old BALB/c mice [61]. The cleared mammary fat pads were then isolated when the mice reached 9 wks of age.

Mouse mammary tumors were generated by implanting mammary tumor cells into the #4 mammary fat pad of strain-matched 6-8 week-old female mice and then harvesting tumors at 0.5-1cm diameter. For the 4T1 model which is derived from a spontaneous mammary tumor arising in a retired BALB/c breeder [62], 40,000 cells were injected and tumors were harvested after 14 days. For the F3II model, also derived from a spontaneous mammary tumor in a BALB/c mouse [63], 500,000 cells were injected and tumors were harvested after 11 days. For the MVT1 model which is a cell line derived from a mammary tumor arising in a MMTV-Myc/VEGF bitransgenic FVB/N mouse [64], 200,000 cells were injected and tumors were harvested after 28 days. Tumors were rinsed in PBS and bisected. Half was snap-frozen and stored in liquid N_2_ for TGF-β extraction, while the other half was fixed in 10% neutral buffered formalin overnight for *in situ* hybridization and immunostaining. To address the possible contribution of TGF-β in the blood to the measured TGF-β levels in normal spleen, lung and liver or the 4T1 tumors, tissues were harvested directly from 4-5 mice and compared with tissues harvested from 4-5 additional mice following cardiac perfusion of the mice with PBS to remove residual blood [65]. Blood was collected by retro-orbital puncture and platelet-poor plasma was prepared as previously described [38].

### Acid-ethanol extraction of frozen tissues

Acid-ethanol extraction was modified from the method of Roberts et al [16]. Frozen tissues (ideally ~100 mg) were weighed, placed into Precellys® reinforced tubes (2 mL) containing 2.8 mm ceramic beads (CK28-R) (Bertin Technologies #KT-03961-1-002.2) and put on dry ice until use. RIPA lysis buffer (Millipore #20-188) containing HALT protease inhibitor cocktail (Thermo Scientific #78440) was added (6 mL/g tissue) and samples were homogenized in a Precellys® 24 homogenizing system with Cryolys cooling at 4°C at 5500 rpm (2 × 20 sec). The Precellys® system allows reproducibly high efficiency, rapid homogenization of multiple samples simultaneously under controlled temperature conditions. In some instances tissues were homogenized into T-PER Tissue Extraction Reagent (Life Technologies #78510) or 50 mM Tris-HCl (pH 7.5). For acid-ethanol extraction of fresh tissue or RIPA extracts, samples were put on ice and transferred to 15 mL tubes containing 6 mL acid-ethanol/mL RIPA extract or 6 mL acid-ethanol/gram of fresh tissue. Acid-ethanol solution contains 60 ml 95% ethanol, 30 mL H_2_O, 1.2 ml conc. HCl, 6 mg phenylmethylsulfonyl fluoride (Sigma-Aldrich #P7627) and 0.3 mg Pepstatin A (Sigma-Aldrich #P5318). Samples were rocked overnight at 4°C. Extracts were centrifuged at 3500g for 10 min and the supernatants were transferred to pre-rinsed MWCO 3,500 Spectra/Por6 dialysis tubing (Spectrum Labs #132590). Samples were dialyzed against 4 mM HCl (3 × 100 vol) at 4°C over 72 h and then transferred to tubes and centrifuged at 3500g for 10 min. Supernatants were transferred to polypropylene 15 ml conical tubes which had been pre-treated with the siliconizing agent Sigmacote (Sigma Aldrich #SL2-100) to minimize losses of TGF-β to the tube walls, and frozen. Frozen samples were lyophilized to dryness, dissolved in 400 μl assay buffer (4mM HCl/150 mM NaCl/0.1% crystalline BSA (Sigma-Aldrich cat # 05470)), transferred to a 1.5 mL microfuge tube pretreated with Sigmacote and stored at −80°C. Prior to assay, samples were clarified by centrifugation at 5000g for 5 min. In practical terms, we found the minimum weight of starting tissue that could be reliably processed through all the preceding steps was 20 mg. Plasma samples were assayed directly without acid-ethanol extraction.

To determine the stability of the tissue extracts, following acid-ethanol extraction, dialysis, and lyophilization, extracts were dissolved in assay buffer, placed in siliconized microfuge tubes and stored at −80°C. Supplementary Figure S1A shows that 4-5 freeze-thaw cycles for reconstituted extracts from an MVT1 tumor or mouse mammary gland did not significantly change the TGF-β levels. Additionally, extracts stored for 10 months showed quantities of TGF-β1 and 3 within 10% and 25%, respectively, of those determined initially. The lower levels of TGF-β2 from these tissues appeared to be less stable upon storage, with the TGF-β2 levels decreasing approximately 40% during storage.

### Quantitation of TGF-β 1, 2 and 3 in tissue extracts and plasma

TGF-β isoforms were quantitated using the TGF-β Premixed Luminex Performance Assay kits from R&D Systems, in either filtration (#FCST17) or magnetic (#FCSTM17) formats. Tissue extracts were generally diluted 1:5 with RD5-49 buffer (supplied with the kit) while plasma samples were activated with HCl, neutralized and diluted in RD6-50 buffer according to the manufacturer's instructions. Our initial studies were done using a filtration-based Luminex assay for TGF-β1, 2 and 3 from R&D Systems followed by detection on the Bio-Plex 200 system. When a magnetic-based assay became available from R&D Systems we changed to this platform based on its increased ease of use and lower background. Detection for the magnetic-based assay was performed on a Bio-Plex MAGPIX reader. We compared TGF-β1, 2 and 3 levels of 15 samples in both assay formats, using samples from tumors and normal mouse tissues to cover the widest possible range of TGF-β levels. Supplementary Figure S1B shows the correlation coefficient is >0.95 for each of the isoforms suggesting that the two platforms yield comparable results. Antibody cross-reactivity was evaluated by assaying 25-90 ng/ml (2x the upper detection limit of the assay) recombinant TGF-β 1, 2 or 3 in the absence of other isoforms. TGF-β levels in tissues were normalized to the weight of the tissue sample extracted and are expressed as ng TGF-β/g tissue. Reported tissue levels of TGF-β proteins derived from measurements on acid-ethanol extracts are corrected for the 50% sample loss we have observed in processing the acid-ethanol extracts which was determined by addition of trace ^125^I-labeled TGF-β1 to parallel samples prior to extraction [18].

### Correlation of mRNA and protein levels

As part of a different project, we performed transcriptomic analysis of four primary tumors each from twelve different models of metastatic mammary cancer using the Affymetrix Mouse 1.0ST array (unpublished data, GEO accession number: GSE69006). Here we performed acid-ethanol extraction and TGF-β protein isoform quantitation on 5 additional tumors for each of the tumor models. We compared the median Affymetrix mRNA signal for a given TGF-β isoform and tumor model with the median isoform-specific TGF-β protein level for the same model.

### In situ hybridization

RNA expression *in situ* was determined with the Advanced Cell Diagnostic's [ACD] (Hayward, CA) Pretreatment Kit (cat# 310020) for formalin-fixed, paraffin-embedded (FFPE) tissue and RNAScope® 2.0 HD Assay-Brown kit (cat# 310035), as per the manufacturer's instructions. Probes specific for the mouse genes Tgfβ1 (cat# 407751, gene accession # NM_011577.1), Tgfβ2 (cat# 406181, accession # NM_009367.3), Tgfβ3 (cat# 406211, accession # NM_009368.3), the housekeeping gene Ppib (cat# 313911, accession # NM_011149.2), or the negative control bacterial gene Dapb (cat# 310043, accession # EF191515) were designed and produced by ACD. For each target RNA the signal was detected following incubation with 3,3′-Diaminobenzidine (DAB) substrate and subsequent washes with water. The nuclei were counterstained with 50% solution of Gill's Hematoxylin #1, washed with water, and blued with 0.02% ammonia water.

### Immunohistochemistry

Sections of normal mammary gland and mammary tumors were stained for intracellular TGF-β1, TGF-β2 and TGF-β3 as described previously [52].

### Data analysis and statistics

Data analysis and graphing were done using GraphPad Prism 6. Values are reported as mean ± standard deviation from at least 4 samples. Significance was determined by the unpaired t-test when comparing two groups and by one-way ANOVA with Tukey's multiple comparison test when comparing multiple groups. P values < 0.05 were considered statistically significant. For visualization of the effects of tissue, mouse strain or gender on TGF-β1 and TGF-β2 levels on a global scale, data were imported into Partek® Genomics Suite 6.5, where scatter plots of individual data points were generated and centroids for the factor variable under comparison were calculated and displayed.

## SUPPLEMENTARY FIGURES AND TABLES


