# Supplementary material for: Quantitation of TGF-β proteins in mouse tissues shows reciprocal changes in TGF-β1 and TGF-β3 in normal vs neoplastic mammary epithelium
Source: Oncotarget. 2016 May 17;7(25):38164–79. doi: 10.18632/oncotarget.9416 (PMC5122380; doi:10.18632/oncotarget.9416)
Supplement: Supplementary file 1 [file oncotarget-07-38164-s001.pdf]

SUPPLEMENTARY FIGURES AND TABLES

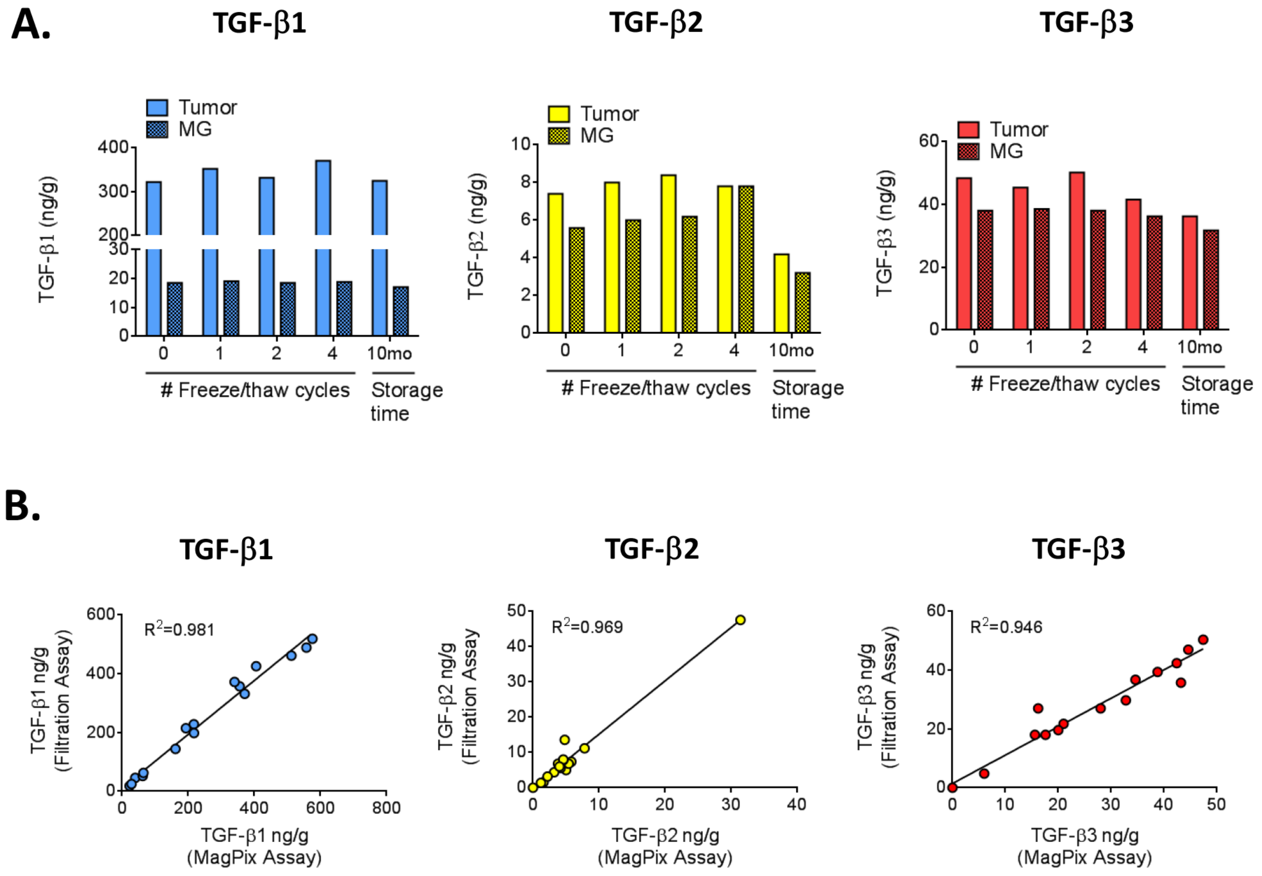

Supplementary Figure S1: Characterization of TGF-β isoform multiplex assay. A. TGF-β isoform levels were measured in RIPA/acid-ethanol extracts of MVT1 mammary tumor (solid bars) or BALB/c mammary gland (MG, cross-hatched bars) which had been stored at −80°C and frozen/thawed for the number of times indicated. **B.** Fifteen tissue extracts from mouse kidneys, mammary glands and mammary tumors were assayed in both the filtration and the magnetic versions of the R&D System TGF-β isoform Luminex kit. Values for each isoform of the samples are plotted and the correlation coefficient (R^2) was calculated.

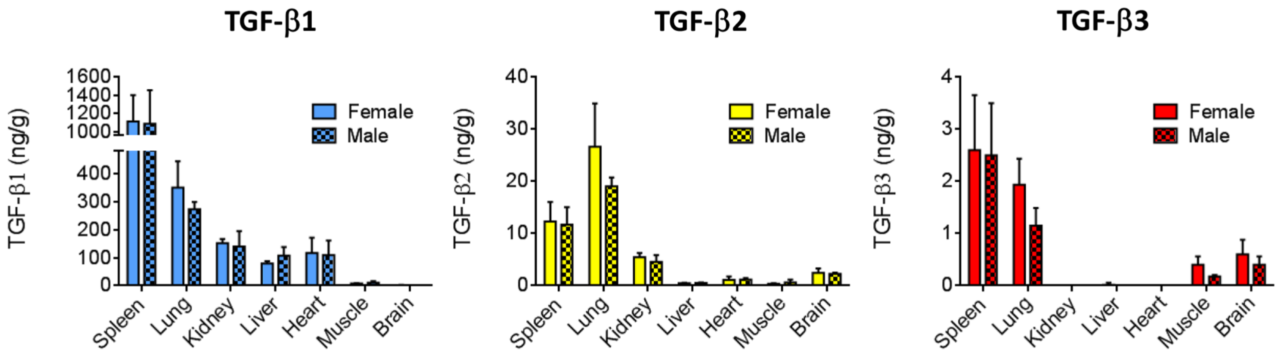

Supplementary Figure S2: Comparison of TGF-β isoform levels in male and female mice. TGF-β isoform levels were measured in RIPA/acid-ethanol extracts of tissues from adult male and female FVB/N mice. Results are mean +/- SD (n=4). There were no statistically significant differences between males and females for any of the tissues analyzed.

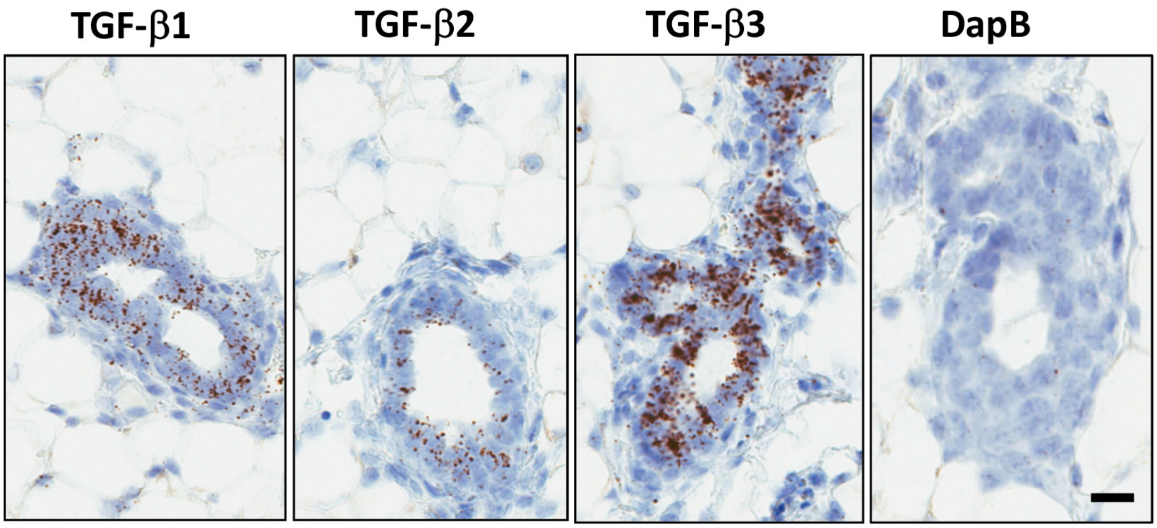

Supplementary Figure S3: TGF-β isoform in situ hybridization in FVB/N mammary gland. In situ hybridization using probes for the indicated molecules in a virgin mammary gland from an adult FVB/N mouse. Brown dots indicate positive signal. DapB was used as a negative control. Bar = 25 µm

Supplementary Table S1: Comparison of TGF-β1 protein levels in tissues from different mouse strains

Tissue	BALB/C	FVB/N	C57BL/6	129S1	p value[b]
Spleen	920.8 ± 402[a]	1085 ± 243	1328 ± 182	885. ± 81.4	ns[c]
Lung	405.6 ± 133	348.8 ± 92.8	222.4±101.4	226.6 ± 89	ns
Kidney	91.8 ± 37.4*	**151.6±15*^**	114.2 ± 18.4	83.8 ± 22.4^	*p<0.05,^p<0.01
Liver	88.2 ± 46.4	79.0 ± 8.4	94.8 ± 72.2	82.2 ± 30.4	ns
Mam Gland	**80.4 ± 33.2***	54.2 ± 28.2	74.6 ± 38.6	16.4 ± 5.2*	*p<0.05
Uterus	42.4 ± 17.0	45.2 ± 11.2	36.0 ± 7.8	31.2 ± 14.2	ns
Heart	36.0 ± 18.0*	**115.8±55.0***	69.0 ± 5.8	58.6 ± 8.8	*p<0.05
Muscle	4.4 ± 0.6	7.6 ± 0.4	7.6 ± 2.6	5.2 ± 1.6	ns
Brain	2.4 ± 2.2	2.2 ± 0.8	2.8 ± 1.4	1.2 ± 0.8	ns

TGF-β1 protein levels were measured in RIPA/acid-ethanol extracts from adult female mice of the indicated strains. Results are mean ± SD (n=4). Units are ng/g tissue. Where values differ significantly, the strain showing the highest level of TGF-β1 for a tissue is highlighted in bold.

[a]*Mean ± Standard Deviation*

[b]*One-way ANOVA with Tukey's multiple comparison test comparing all four strains with each other. P-values for indicated pairwise comparisons that show statistically significant differences.*

[c]*Not significant*

Supplementary Table S2: Comparison of TGF-β2 protein levels in tissues from different mouse strains

Tissue	BALB/C	FVB/N	C57BL/6	129S1	p value[b]
Spleen	4.6 ± 2.4[a]^	**12.4 ± 3.0*^**	7.4 ± 1.0*	8.4 ± 1.0	*p<0.05,^p<0.001
Lung	11.6 ± 6.8*	**26.6 ± 8.4*^**	14.4 ± 6.6	9.8 ± 4.0^	*p<0.05,^p<0.01
Kidney	3.0 ± 0.6*	**2.7 ± 0.4*^#**	3.2 ± 0.4^	2.4 ± 0.2[#]	*^p<0.001,[#]p<0.0001
Liver	0.6 ± 0.2	0.4 ± 0.2	0.6 ± 0.2	0.6 ± 0.2	ns[c]
Mam Gland	3.8 ± 2.2*	**9.4 ± 3.6*^**	3.8 ± 3.0	0.8 ± 0.6^	*p<0.05,^p<0.005
Uterus	4.6 ± 1.4	**5.4 ± 2.2***	2.6 ± 1.0	2.0 ± 1.2*	*p<0.05
Heart	0.8 ± 0.4	1.2 ± 0.6	1.0 ± 0.4	2.0 ± 1.4	ns
Muscle	0.2 ± 0	0.4 ± 0.2	0.4 ± 0.2	0.4 ± 0.2	ns
Brain	1.6 ± 0.6	2.4 ± 0.8	**2.8 ± 0.4***	1.4 ± 0.8*	*p<0.05

TGF-β2 protein levels were measured in RIPA/acid-ethanol extracts from adult female mice of the indicated strains. Results are mean ± SD (n=4). Units are ng/g tissue. Where values differ significantly, the strain showing the highest level of TGF-β2 for a tissue is highlighted in bold.

[a]*Mean ± Standard Deviation*

[b]*One-way ANOVA with Tukey's multiple comparison test comparing all four strains with each other. P-values for indicated pairwise comparisons that show statistically significant differences.*

[c]*Not significant*

Supplementary Table S3: Comparison of TGF-β3 protein levels in tissues from different mouse strains

Tissue	BALB/C	FVB/N	C57BL/6	129S1	p value[b]
Spleen	2.8 ± 1.2[a]	2.6 ± 0.8*	2.6 ± 0.4	**4.2 ± 0.4***	*p<0.05
Mam Gland	51.6 ± 18.6*	**69.8 ± 33.2^**	31.6 ± 14.2	8.2 ± 2.6*^	*p<0.05,^p<0.01

TGF-β3 protein levels were measured in RIPA/acid-ethanol extracts from adult female mice of the indicated strains. Results are mean ± SD (n=4). Units are ng/g tissue. Where values differ significantly, the strain showing the highest level of TGF-β3 for a tissue is highlighted in bold

[a]*Mean ± Standard Deviation*

[b]*One-way ANOVA with Tukey's multiple comparison test comparing all four strains with each other.*

P-values for indicated pairwise comparisons that show statistically significant differences.

Supplementary Table S4: Comparison of TGF-β isoform levels in male and female mice

	TGF-β1	TGF-β1	TGF-β2	TGF-β2
	**Female**	**Male**	**Female**	**Male**
Spleen	1085.4 ± 243	1111.4 ± 291	12.4 ± 3	11.8 ± 3.4
Lung	348.8 ± 92.8	272.2 ± 25.8	26.2 ± 8.4	19.0 ± 1.8
Kidney	151.6 ± 15.0	139.4 ± 54.4	5.4 ± 0.8	3.2 ± 0.4
Liver	79.0 ± 8.4	107.2 ± 30.0	0.4 ± 0.2	0.6 ± 0.2
Heart	115.8 ± 55.0	108.8 ± 51.8	1.2 ± 0.6	1.2 ± 0.4
Muscle	7.6 ± 0.4	10.4 ± 5.6	0.4 ± 0.2	0.4 ± 0.2
Brain	2.2 ± 0.8	1.8 ± 0.4	2.4 ± 0.8	2.4 ± 0.2

TGF-β isoform levels were measured in RIPA/acid-ethanol extracts of tissues from adult male and female FVB/N mice. Results are mean +/- SD (n=4). Units are ng/g tissue. There are no statistically significant differences between males and females in any isoform for any of the tissues examined (Students t-test).
